# Lingering Hesitancy: Persistent Uncertainty About the COVID-19 Vaccines Among Previously Vaccinated Individuals

**DOI:** 10.1016/j.focus.2025.100437

**Published:** 2025-09-05

**Authors:** Francis K. Kazungu, Sinan Almukhtar, Emily Stiehl, Manorama M. Khare, Ronald C. Hershow, Sanjib Basu, Noah McWhirter, Sage J. Kim

**Affiliations:** 1 Division of Health Research and Evaluation, Department of Family and Community Medicine, University of Illinois College of Medicine Rockford, Rockford, Illinois; 2 Division of Epidemiology and Biostatistics, School of Public Health, University of Illinois Chicago, Chicago, Illinois; 3 Division of Health Policy and Administration, School of Public Health, University of Illinois Chicago, Chicago, Illinois

**Keywords:** Lingering hesitancy, COVID-19 vaccine trust, vaccine hesitancy, vaccine attitudes, vaccine messaging, routine vaccination

## Abstract

**Introduction:**

Routine vaccination is an important public health measure to prevent severe illness from COVID-19. Although the determinants of vaccine hesitancy have been explored in prior research, limited information is available on the varying perceptions of the COVID-19 vaccines among previously vaccinated individuals. This study investigates how individuals previously vaccinated against COVID-19 might develop hesitancy toward future doses, a concept that the authors define as lingering hesitancy.

**Methods:**

The authors conducted an online cross-sectional survey with 560 vaccinated respondents in 6 urban Chicago community areas (*n*=440) and Stephenson County, a rural county in Northern Illinois (*n*=120), between December 2021 and April 2022. The authors explored the role of an individual’s sociocultural context (i.e., urban/rural setting, political affiliation, and race/ethnicity) in developing lingering hesitancy. Lingering hesitancy was operationalized using 2 constructs: (1) negative COVID-19 vaccine attitudes and (2) COVID-19 vaccine safety concerns. The authors also investigated whether mistrust in the government and institutions as well as barriers to accessing the COVID-19 vaccines mediate the relationship between context and lingering hesitancy.

**Results:**

The authors found significantly higher negative attitudes toward the COVID-19 vaccines and higher safety concerns among conservative versus liberal respondents (b=0.37, *p*<0.001; b=0.21, *p*<0.01, respectively) and among urban versus rural residents (b=0.45, *p*<0.001; b=0.37, *p*<0.001). Black/African American participants reported higher vaccine safety concerns than White participants (mean=2.6 vs 2.4, *p*<0.05). A significant portion of lingering hesitancy among conservative and Black/African American respondents was mediated by mistrust in the government and institutions. Barriers partially mediated vaccine safety concerns among Hispanic respondents.

**Conclusions:**

Most public health efforts aimed at COVID-19 vaccine outreach and messaging have focused on increasing vaccine uptake, thereby targeting unvaccinated individuals. As the focus shifts toward annual COVID-19 vaccinations, it is essential to consider lingering hesitancy among those who have received previous vaccine doses. This article provides a framework for implementing vaccine-messaging campaigns that increase confidence and build trust in routine vaccinations.

## Highlights

• Previously vaccinated individuals have varying perceptions of the COVID-19 vaccines.
• Persistent concerns about a vaccine could influence future vaccination decisions.
• Sociocultural context plays a role in the development of lingering hesitancy.
• Mistrust mediates the relationship between context and lingering hesitancy.
• Trusted messengers are essential for boosting confidence in routine vaccinations.

## INTRODUCTION

Although routine vaccination is an important public health measure to prevent serious illness from coronavirus disease 2019 (COVID-19), the uptake of booster doses for the COVID-19 vaccine has been steadily declining. As of July 2024, a total of 80.7% of the U.S. adult population had received at least 1 dose of the COVID-19 vaccine.^1^ However, only 22.3% had received the updated 2023–2024 vaccines, and 42.1% did not intend to get these updated 2023–2024 vaccines.^1^ COVID-19 vaccination rates also varied substantially across subpopulations. In Chicago, a large urban city, 57.3% of Black residents completed the primary series (i.e., 2 doses of Moderna/Pfizer or 1 shot of Johnson & Johnson vaccine) compared with 69.9% of White and 72.1% of Hispanic Chicagoans.^2^ The uptake of the 2023–2024 COVID-19 vaccine remained low: 10.9% for Black, 23.6% for White, and 10.9% for Hispanic Chicagoans. In Stephenson County, a rural county in Northern Illinois, an estimated 62% of residents received the primary series of vaccines^3^ and only 12.2% had taken the updated 2023–2024 vaccines as of July 2024.^4^ The tepid uptake of booster doses suggests a possible lingering uncertainty about the COVID-19 vaccine, even among individuals who received an initial dose.

Previous studies document factors associated with COVID-19 vaccine hesitancy among unvaccinated adults. These factors include concerns about side effects, long-term health implications, the effectiveness of the vaccine, and a lack of trust in institutions developing and distributing the vaccine.^5^, ^6^, ^7^ Contextual factors, such as political affiliation, have also been associated with vaccine hesitancy.^8^^,^^9^ However, less is known about vaccine attitudes among individuals who received an initial dose of the COVID-19 vaccine but did not receive subsequent doses. Research on other vaccines with multiple doses, including the human papillomavirus vaccine, suggests that a lack of knowledge about the purpose or number of doses in the series,^10^ miscommunication with providers about scheduling and timing of the follow-up visit, or barriers to access (such as a lack of insurance) can affect series completion.^10^, ^11^, ^12^

During the COVID-19 pandemic, public health officials in Chicago made substantial efforts to address known barriers, including access, knowledge, and trust.^13^ Despite a multitude of informational materials and improved access to the vaccines, uptake among previously vaccinated individuals was lower than expected. COVID-19 vaccination is now recommended annually.^14^^,^^15^ Therefore, it is important to understand the perceptions of the COVID-19 vaccine among people who have received primary doses but have not taken the boosters.

In this article, the authors explore lingering hesitancy, which is defined as an individual’s concerns or uncertainty about a vaccine that persist or increase after receiving an initial dose. This is a distinct concept from regular vaccine hesitancy, which often refers to individuals who remain unvaccinated. In this analysis, the authors explore the role of an individual’s context (i.e., urban versus rural setting, political affiliation, and race/ethnicity) in developing lingering hesitancy. The authors conceptualize lingering hesitancy as safety concerns and negative attitudes toward the vaccines. The authors aim to understand the factors associated with lingering hesitancy among individuals who received at least 1 dose of the COVID-19 vaccine and explore potential mediating effects that may influence the relationship between context and lingering hesitancy. Building on the literature around human papillomavirus vaccines, the authors hypothesize that mistrust will mediate the relationship between context and safety concerns, such that a higher level of mistrust will be associated with greater safety concerns. The authors also expect that barriers will mediate the relationship between an individual’s context and negative attitudes about the vaccine, such that the harder it is to access the vaccine, the more negative people’s perceptions of the vaccine will be.

## METHODS

The authors conducted an online cross-sectional survey in 6 urban Chicago community areas and Stephenson County, a rural county in Northern Illinois, between December 2021 and April 2022. The study protocol was approved by the University of Illinois Chicago IRB (IRB#: 2021-0737).

**Figure 1:**
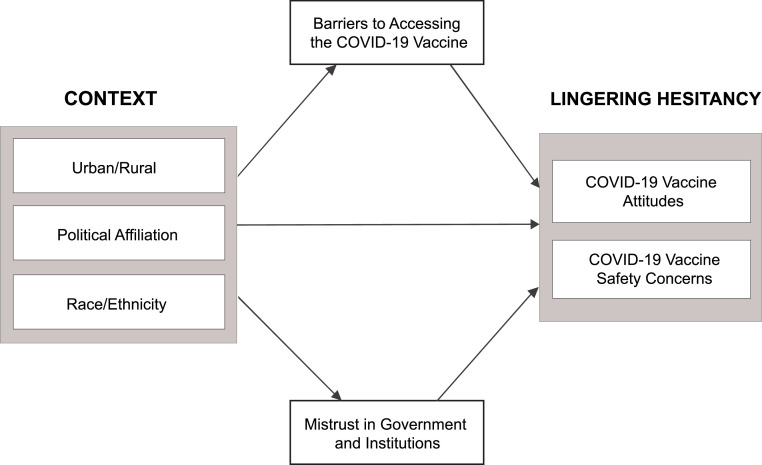
Proposed model of the contextual factors and potential mediators affecting lingering hesitancy.

The conceptual model of this study is shown in Figure 1. Drawing from previous literature on vaccine hesitancy,^5^^,^^9^^,^^16^, the authors highlight the role of context within which previously vaccinated individuals might develop lingering hesitancy. To operationalize lingering hesitancy, the authors used 2 constructs (defined in more detail under the Measures section): (1) individual COVID-19 vaccine attitudes, including perceptions of the imminent risk of contracting COVID-19 and knowledge/beliefs regarding how the vaccine works and (2) concerns about COVID-19 vaccine safety and effectiveness. Furthermore, the authors explored whether mistrust in the government, institutions, and vaccine manufacturers as well as perceived barriers to accessing the COVID-19 vaccine mediate the relationship between context (i.e., rural versus urban setting, political affiliation, and race/ethnicity) and lingering hesitancy.

### Study Sample

The research team collaborated with a rural local health department (LHD), community-based organizations (CBOs), and citizen scientists to recruit survey participants in communities with low vaccine uptake. These communities were identified as high need during the COVID-19 pandemic and received additional outreach regarding the vaccine. The authors also recognize that people in communities with low vaccine uptake might be exposed to information that promotes vaccine hesitancy. The citizen scientists were staff of the CBOs and LHD who received training on the foundations of research and evaluation methods. The citizen scientists also worked on COVID-19 vaccine outreach, including providing education about the vaccine and scheduling vaccine appointments through their organizations.^13^ For this study, the citizen scientists were involved in designing survey questions, recruiting study participants, and interpreting study findings. They also assisted the authors in recruiting a convenience sample from their communities to participate in an online survey.

In Chicago, participants were recruited primarily through community events. The citizen scientists distributed flyers with QR codes or emailed a survey link to interested participants. In Stephenson County, the citizen scientists sent emails to those on the contact lists of the Farm Bureau, Emergency Medical Technician service, local college, school district, and local churches.

Eligible participants were adults (aged ≥18 years) who indicated that they could read or communicate in English or Spanish and lived in Chicago or Stephenson County. All respondents provided informed consent and received a $10 gift card for completing the online survey. The authors asked additional questions in the survey to verify eligibility and excluded participants with missing ZIP codes (*n*=40), participants residing outside Chicago or Stephenson County (*n*=134), and responses that did not indicate being recruited by a participating CBO/LHD (*n*=88). Responses with duplicate timestamps (*n*=839)^a^ or <50% complete (*n*=12) were also excluded. Finally, the authors excluded any unvaccinated participants (*n*=65) and restricted cases to fully or partially vaccinated individuals (i.e., those who reported having received at least 1 dose of the COVID-19 vaccine). The final sample included 560 respondents.

### Measures

Survey questions were adapted from other COVID-19 studies, including the Prevention Research Center Vaccine Confidence Network of the Centers for Disease Control and Prevention.^17^

As variables, the survey included questions on attitudes and safety concerns regarding the COVID-19 vaccines, whether participants trusted the government, institutions, and vaccine manufacturers, as well as the perceived barriers to accessing the COVID-19 vaccines. Questions were measured on a 5-point Likert-type scale (from 1=strongly disagree to 5=strongly agree).

The authors conceptualized the dependent variable lingering hesitancy as (1) concerns about COVID-19 vaccine safety and (2) negative attitudes toward the COVID-19 vaccine. A higher score indicates that participants had more concerns about COVID-19 vaccine safety^b^ and had negative perceptions of the COVID-19 vaccine (i.e., seeing it as unnecessary or ineffective).

The authors examine 2 potential mediating variables: (1) mistrust in the government, institutions, and manufacturers of COVID-19 vaccines and (2) barriers to accessing the vaccines. A higher score indicates greater mistrust in the government and institutions. Higher scores also indicate a greater perception of barriers to accessing the vaccines. More specifically, participants reported that they would be less likely to get vaccinated if they encountered obstacles such as showing proof of health insurance, difficulty scheduling an appointment, or experiencing long wait times (Appendix Table 1, available online).

In addition, the survey included contextual variables (i.e., rural–urban residence, political affiliation, and race/ethnicity) and demographic characteristics (self-identified gender, age, income, employment, and education) that were measured as categorical variables.

### Statistical Analysis

The authors performed a factor analysis with varimax rotation to assign variables to the relevant constructs. Four factors were extracted: (1) negative COVID-19 vaccine attitudes, (2) COVID-19 vaccine safety concerns, (3) mistrust in government and institutions, and (4) the perceived impact of barriers to accessing the COVID-19 vaccines. Each factor was internally consistent and well-defined by the variables (Appendix Table 1). Survey items were assigned to a construct based on the highest loading, with a minimum cut off point of 0.40. Two items *(I do not have to get vaccinated because others around me are vaccinated against COVID-19* and *I trust information and decisions about COVID-19 provided by my local health department*) were complex variables and were assigned to final constructs based on the conceptual model of this study. The authors excluded 2 additional items because they did not load on any factor.

Inter-item correlations with a Cronbach Coefficient α of >0.60 were retained for each construct. Furthermore, all interdomain correlations were <0.70, providing evidence that each group of variables measured a distinct construct (Appendix Table 2, available online). Factor scores were calculated for each participant and were used for downstream analysis.

The authors characterized respondents by context (i.e., rural–urban residence, political affiliation, and race/ethnicity^c^) and by their willingness to receive a COVID-19 booster shot. The authors compared mean factor scores across the contextual variables, applying the Tukey–Kramer adjustment for multiple group comparisons. To show the variation in responses within the vaccinated sample in this study, the authors summarized individual survey items by the percentage of participants who picked each response. Significant differences were assessed using the chi-square test of independence.

Multiple linear regression was used to explore associations between the contextual variables and lingering hesitancy (i.e., negative COVID-19 vaccine attitudes and COVID-19 vaccine safety concerns). To assess the impact of each potential mediator (i.e., mistrust in government and institutions, and perceived barriers to accessing the COVID-19 vaccines), the authors ran models with the contextual variables as predictors and the mediators as outcomes. The authors then examined associations between the contextual variables and lingering hesitancy. Finally, the authors tested the impact of the mediators on lingering hesitancy, accounting for the effect of the contextual variables. All tests were 2-tailed, with a significance level of 0.05, and the data were analyzed using SAS Enterprise, version 9.4.^18^

## RESULTS

Participant characteristics are shown in Table 1. The study included a final sample of 560 responses, with 440 from Chicago (urban setting) and 120 from Stephenson County, Illinois (rural setting).

**Table 1:** Demographic Characteristics of Urban and Rural Participants

Characteristics	Urban participants (Chicago)	Rural participants (Stephenson County)
	*n* (%) (*n*=440)	*n* (%) (*n*=120)
Age, years***		
18–34	191 (43.7)	26 (21.7)
35–54	221 (50.6)	60 (50.0)
55–64	18 (4.1)	25 (20.8)
65+	7 (1.6)	9 (7.5)
Gender*		
Female	257 (58.5)	87 (72.5)
Male	172 (39.2)	32 (26.7)
Transgender or nonbinary	10 (2.3)	<1%
Race/ethnicity***		
White	165 (38.0)	103 (85.8)
Hispanic	158 (36.4)	9 (7.5)
Black/African American	79 (18.2)	7 (5.8)
Other race (including Native American/American Indian/Alaska Native, Native Hawaiian/Pacific Islander, Asian, and ≥2 races)	32 (7.4)	<1%
Education***		
≥Bachelor’s degree	184 (43.7)	83 (69.7)
<Bachelor’s degree	237 (56.3)	36 (30.3)
Employment status*		
Employed full-time	328 (79.0)	104 (89.7)
Employed part-time	60 (14.5)	10 (8.6)
Unemployed	27 (6.5)	2 (1.7)
Annual income, $***		
<35,000	64 (15.0)	14 (11.7)
35,001–50,000	114 (26.8)	18 (15.0)
50,001–75,000	130 (30.5)	24 (20.0)
>75,000	84 (19.7)	55 (45.8)
Preferred not to answer	34 (8.0)	9 (7.5)
Political affiliation		
Liberal	176 (43.9)	39 (33.3)
Conservative	84 (21.0)	29 (24.8)
Moderate	89 (22.2)	30 (25.6)
Preferred not to answer	52 (13.0)	19 (16.3)

*Note:* A total of 560 participants were included in this study. Statistical significance at *p<0.05, **p<0.01, and ***p<0.001. Significant differences among groups were tested using the χ^2^ test of independence.

Most respondents were female (61.5%), aged 35–54 years (50.4%), and employed full-time (81.3%). As of April 2022, a total of 95.9% of the study sample had received the primary series of vaccines (i.e., 2 shots of Moderna/Pfizer or 1 shot of Johnson & Johnson vaccine) and 4.1% had received only the initial dose of Moderna or Pfizer. In addition, 76% (*n*=422) of respondents were willing to receive a booster, 14.1% were unwilling, and 9.8% were unsure (Appendix Table 3, available online).

A greater proportion of participants in the urban than in the rural setting were aged 18–34 years (43.7% vs 21.7%, *p*<0.001), identified as Hispanic (36.4% vs 7.5%, *p*<0.001), and identified as Black or African American (18.2% vs 5.8%, *p*<0.001). Proportionately, more rural than urban participants had a bachelor’s degree or higher (69.7% vs 43.7%, *p*<0.001) and income >$75,000 (45.8% vs 19.7%, *p*<0.001). There was no statistically significant difference in political affiliation between the urban and rural participants.

Descriptive statistics (means and SDs) for the key survey constructs are summarized in Table 2. The authors report how perceptions of the COVID-19 vaccines differ within each construct as follows.

**Table 2:** Unadjusted Mean Scores of Survey Constructs by Residence, Political Affiliation, Race/Ethnicity, and Intention to Take a COVID-19 Booster

	Mediators		Outcomes	
Contextual variables	Mistrust, mean (SD)	Barriers, mean (SD)	Negative vaccine attitudes, mean (SD)	Vaccine safety concerns, mean (SD)
Full sample (N=560)	2.7 (0.9)	3.1 (0.8)	2.2 (0.8)	2.5 (0.7)
Residence				
Rural	2.6 (0.8)	3.1 (0.9)	1.9^a^ (0.8)	2.2^a^ (0.7)
Urban	2.7 (0.9)	3.1 (0.7)	2.3^a^ (0.8)	2.5^a^ (0.7)
*p*	0.517	0.943	**<0.001**	**<0.001**
Political affiliation				
Conservative	2.8a,b (0.9)	3.0^a^ (0.7)	2.6a,b,c (0.8)	2.5^a^ (0.7)
Liberal	2.5a,c (0.8)	2.9^b^ (0.8)	2.0^a^ (0.8)	2.3a,b,c (0.7)
Moderate	2.6^b^ (0.9)	3.2 (0.7)	2.1^b^ (0.7)	2.5^b^ (0.7)
Preferred not to answer	2.8^c^ (0.9)	3.4a,b (0.9)	2.0^c^ (0.8)	2.6^c^ (0.8)
*p*	**<0.01**	**<0.001**	**<0.001**	**<0.01**
Race/ethnicity*				
Black	2.5 (0.8)	3.0^a^ (0.6)	2.1 (0.7)	2.6^a^ (0.6)
White	2.7 (0.8)	3.0^b^ (0.9)	2.3^a^ (0.8)	2.4^a^ (0.7)
Hispanic	2.7 (0.9)	3.2a,b (0.9)	2.1^a^ (0.9)	2.5 (0.7)
*p*	0.160	**<0.01**	**<0.05**	**<0.05**
Intention to take a booster				
Willing	2.5^a^ (0.8)	3.0^a^ (0.8)	2.0^a^ (0.7)	2.3^a^ (0.7)
Not willing	3.5^a^ (0.8)	3.1 (0.7)	3.1^a^ (0.7)	3.0^a^ (0.6)
Not sure	3.2^a^ (0.7)	3.3^a^ (0.8)	2.4^a^ (0.7)	2.7^a^ (0.5)
*p*	**<0.001**	**<0.05**	**<0.001**	**<0.001**

*Note:* Boldface indicates statistical significance at the corresponding *p*-value. Mean scores for all constructs ranged from 1 to 5. Differences in means were tested using 1-way ANOVA with least-squares adjustment, and the Tukey–Kramer adjustment was applied to account for multiple group comparisons (data were assessed for normality using the Shapiro–Wilk test and visual inspection of histograms and Q-Q plots).

a,b,c Means with the same superscript letter in each subsection are significantly different (e.g., the mean mistrust score for conservative respondents was significantly higher than that for liberal and moderate respondents, and the mean barriers score for Hispanic respondents was significantly higher than that for Black and White respondents).

⁎ Participants identifying as Asian, Native American/American Indian, Alaska Native/Hawaiian, or identifying with ≥2 races were excluded due to a small sample size.

Individuals who identified as conservative expressed significantly greater mistrust of the government, institutions, and vaccine manufacturers than those who identified as liberal or moderate (mean=2.8 vs 2.5 and 2.6, *p*<0.01; Table 2). Interestingly, the analysis of single survey items showed that a greater proportion of rural participants (78.8%) than participants in the urban setting (54.0%) trusted their LHD (chi-squared=23.6, df=2, *V*=0.2, *p*<0.001; Appendix Table 4, available online).

A more significant impact of barriers was reported among Hispanic respondents compared with Black and White respondents (mean=3.2 vs 3.0 and 3.0, *p*<0.01; Table 2).

The authors operationalized lingering hesitancy as 2 constructs: (1) negative COVID-19 vaccine attitudes and (2) COVID-19 vaccine safety concerns.

The authors found significantly higher negative attitudes toward the COVID-19 vaccines among conservative respondents than among liberal respondents (mean=2.6 vs 2.0, *p*<0.001; Table 2) and among participants living in an urban setting compared with among those in a rural setting (mean=2.3 vs 1.9, *p*<0.001). Individual survey items showed that more urban participants than rural participants reported a workplace mandate for the COVID-19 vaccines (69.3% vs 33.3%, chi-squared=51.3, df=1, *V*=0.3, *p*<0.001; Appendix Table 5, available online).

Significantly higher safety concerns were reported among Black participants than among White participants (mean=2.6 vs 2.4, *p*<0.05) and among urban participants compared with rural participants (mean=2.5 vs 2.2, *p*<0.001). A greater proportion of Black respondents compared with Hispanic and White respondents (58.3% vs 34.6% and 30.9%) indicated that the COVID-19 vaccines were developed too quickly (chi-squared=21.4, df=4, *V*=0.1, *p*<0.01; Appendix Table 4). About a third of the Black respondents (34.9%) also expressed that there was not enough data about the safety of the COVID-19 vaccines (Appendix Table 4).

The authors ran multiple regression models to explore the impact of mistrust, barriers, and contextual factors on lingering hesitancy (i.e., negative COVID-19 vaccine attitudes and COVID-19 vaccine safety concerns). First, the authors found significant associations between the contextual variables and proposed mediators (Table 3, Models 1 and 2). For example, there was significantly greater mistrust of the government, institutions, and vaccine manufacturers among conservative than among liberal respondents (b=0.20, *p*<0.05; Table 3, Model 1) and among individuals who were unwilling or unsure about taking the COVID-19 booster shots than among those who were willing (b=1.07 and 0.72, *p*<0.001; Model 1). Hispanic versus White and moderate versus liberal participants reported significantly higher barriers (b=0.19 and 0.22, *p*<0.05; Model 2).

**Table 3:** Multiple Regression Analysis Measuring the Impact of Contextual Variables on Lingering Hesitancy

	Mediators		Outcomes			
Contextual variables	1^a^ Mistrust	2 Barriers	3 Negative vaccine attitudes	4 Negative vaccine attitudes (mistrust)	5 Vaccine safety concerns	6 Vaccine safety concerns (mistrust)
Intercept	2.45*** (2.32, 2.59)	2.87*** (2.74, 3.01)	2.20*** (2.08, 2.32)	1.12*** (0.92, 1.31)	2.33*** (2.21, 2.45)	1.92*** (1.70, 2.14)
Residence						
Rural	–0.01 (–0.27, 0.08)	–0.01 (–0.19, 0.16)	–0.45*** (–0.60, –0.30)	–0.40*** (–0.53, –0.27)	–0.37*** (–0.51, –0.22)	–0.35*** (–0.50, –0.20)
Political affiliation						
Conservative	0.20* (0.01, 0.39)	0.15 (–0.03, 0.34)	0.37*** (0.21, 0.54)	0.27*** (0.13, 0.42)	0.21** (0.05, 0.37)	0.18* (0.02, 0.34)
Moderate	–0.04 (–0.02, 0.41)	0.22* (0.04, 0.40)	–0.03 (–0.19, 0.13)	–0.03 (–0.16, 0.11)	0.13 (–0.02, 0.28)	0.14 (–0.01, 0.29)
Preferred not to answer	0.19 (–0.27, 0.39)	0.36** (0.14, 0.57)	–0.05 (–0.24, 0.14)	–0.16 (–0.32, 0.01)	0.24* (0.05, 0.42)	0.20* (0.03, 0.38)
Race/ethnicity						
Black/African American	–0.28** (–0.48, –0.07)	–0.01 (–0.31, 0.11)	–0.36*** (–0.53, –0.18)	–0.22** (–0.38, –0.07)	0.10 (–0.07, 0.27)	0.15 (–0.02, 0.32)
Hispanic	–0.10 (–0.27, 0.07)	0.19* (0.01, 0.36)	–0.40*** (–0.55, –0.25)	–0.34*** (–0.47, –0.21)	–0.11 (–0.25, 0.04)	–0.09 (–0.23, 0.05)
Intention to take a booster						
Not willing	1.07*** (0.85, 1.28)	0.12 (–0.09, 0.33)	1.07*** (0.90, 1.26)	0.59*** (0.41, 0.76)	0.60*** (0.42, 0.78)	0.42*** (0.23, 0.62)
Not sure	0.72*** (0.49, 0.96)	0.24 (0.00, 0.48)*	0.51*** (0.30, 0.72)	0.20* (0.02, 0.38)	0.35*** (0.15, 0.55)	0.23* (0.03, 0.43)
Mistrust				0.45***		0.17***
*R^2^*	0.23	0.07	0.34	0.52	0.17	0.20
*p*						
*F*-value	17.98	4.06	29.00	53.86	12.34	13.56

*Note:* Boldface indicates statistical significance (*p<0.05, **p<0.01, ***p<0.001). Values denote the unstandardized β coefficient (with 95% CIs) for each predictor. *Urban, Liberal, White*, and *Willing to take a booster* were set as reference groups for comparison and thus omitted from the table.

a Denotes the model number as referenced in the narrative.

The contextual variables were also associated with lingering hesitancy. The authors found significantly higher negative COVID-19 vaccine attitudes and higher safety concerns among conservative respondents compared with liberal respondents (b=0.37, *p*<0.001, Model 3; b=0.21, *p*<0.01, Model 5), among urban residents compared with rural residents (b=0.45, *p*<0.001, Model 3; b=0.37, *p*<0.001, Model 5), and among individuals who were unwilling or unsure about taking the boosters compared with those who were willing (b=1.07 and 0.51, *p*<0.001, Model 3; b=0.60 and 0.35, *p*<0.001, Model 5).

After accounting for the impact of contextual variables on lingering hesitancy, the authors added mistrust to the models to test for mediation. The authors found that the β coefficients among Black and Hispanic (versus White), rural (versus urban), and conservative (versus liberal) respondents were smaller than in the models without mistrust (comparison of Models 3 versus 4 and 5 versus 6). These findings indicate a potential mediating effect of mistrust on the relationships between the contextual variables and lingering hesitancy. To further examine the mediating effect of mistrust, the authors conducted a bootstrapping analysis to decompose the direct and indirect effects of the contextual variables on lingering hesitancy. The authors found the same mediation pathway as previously described. For example, a significant portion of negative vaccine attitudes and vaccine safety concerns among conservative, Black/African American respondents, and individuals who were unwilling or unsure about taking the boosters was routed through mistrust (Appendix Tables 6 and 7, available online).

The authors also tested for the mediating effect of barriers. Upon adding barriers to the lingering hesitancy models, the association between barriers and negative COVID-19 vaccine attitudes was not significant (Appendix Table 8 [available online], Model 7). However, the relationship between barriers and vaccine safety concerns was positive and significant (b=0.17, *p*<0.001; Appendix Table 8 [available online], Model 9). Bootstrapping analysis further supported these findings. Barriers did not significantly mediate the relationship between context and negative COVID-19 vaccine attitudes (Appendix Table 9, available online). However, a significant portion of vaccine safety concerns among Hispanic respondents and among individuals who identified as politically moderate was routed through barriers (Appendix Table 10, available online).

## DISCUSSION

Routine vaccination against COVID-19 has become an important public health measure to prevent serious illness from the disease. This study shows that some people have persistent concerns and uncertainty about the COVID-19 vaccines even after receiving an initial dose, which could influence future vaccination decisions. This concept, which the authors define as lingering hesitancy, is derived from 2 components: negative COVID-19 vaccine attitudes and COVID-19 vaccine safety concerns.

Recent public health efforts have targeted a “moveable middle” for vaccine uptake, namely, vaccine-hesitant individuals who can be persuaded to vaccinate. This study suggests that people who were once convinced to vaccinate might reassess their decision because of lingering hesitancy. In particular, the authors found that vaccinated individuals with a conservative political affiliation reported relatively higher levels of lingering hesitancy and a greater mistrust of the government, institutions, and vaccine manufacturers. Furthermore, the relationship between conservative political affiliation and lingering hesitancy was mediated by mistrust. These study findings align with previous research showing an association between conservative respondents and higher levels of vaccine hesitancy, potentially caused by a lack of trust in government, institutions, and the news media.^6^^,^^8^^,^^19^

A greater proportion of Black respondents reported vaccine safety concerns, particularly that the vaccine was developed too quickly. These findings are consistent with earlier studies on vaccine hesitancy among Black respondents, associating low vaccine uptake with historical injustices that have eroded trust in research and medical practice.^20^^,^^21^ Despite Black respondents having high safety concerns, they reported more positive COVID-19 vaccine attitudes. Black respondents, in particular, often operate in systems that they do not trust or that are biased against them. Operating in such systems could mean that although Black respondents recognize the value and importance of vaccination to prevent serious illness, they still worry about the development, composition, and safety profile of specific vaccines.^22^ Furthermore, lingering perceptions that a vaccine is unsafe could reduce an individual’s willingness to obtain routine vaccinations even after receiving an initial dose.

In this study, the authors found that rural participants had significantly lower levels of lingering hesitancy than urban participants. Furthermore, vaccinated respondents in rural settings were more likely to trust the information and decisions about COVID-19 provided by their LHD. Although previous literature highlights that rural communities have a relatively greater mistrust of government, institutions, and the medical establishment,^23^^,^^24^ the current study results show that having a strong relationship between rural residents and their LHDs improves trust and could ultimately reduce lingering hesitancy toward subsequent vaccine doses. Simultaneously, urban participants were more likely to report a workplace mandate for the COVID-19 vaccines, which might have negatively affected their perceptions of the vaccine. Other studies on COVID-19 vaccine mandates show that requiring the vaccine in various settings might increase uptake, but does not directly improve vaccine confidence.^25^^,^^26^

Finally, Hispanic respondents (versus Black and White respondents) reported higher barriers to accessing the COVID-19 vaccines, although the barriers construct accounted for limited variance in lingering hesitancy. Current literature highlights the extensive outreach to increase vaccine uptake in Hispanic communities.^27^^,^^28^ Through vaccination clinics, messaging from trusted community leaders, and vaccine recommendations from local healthcare providers,^13^^,^^27^ targeted outreach helped reduce barriers. Such outreach may have also addressed negative attitudes or safety concerns associated with accessibility issues. However, the end of the public health emergency and reductions in funding will decrease access to future COVID-19 vaccines. More work is needed to address these barriers.

Future vaccination campaigns should actively address the specific mistrust of government and institutions within various communities. Although much work during the COVID-19 pandemic was focused on vaccine-hesitant individuals, more efforts should be directed toward providing support and information to persons who previously received the vaccine. Public health officials should continuously provide information about potential side effects, risks, and effectiveness to previously vaccinated individuals. This strategy could alleviate negative attitudes and safety concerns toward the vaccines.^29^ Public health officials could also invest in campaigns that provide training and support for CBOs or healthcare providers^35^ to address specific vaccine safety concerns and build trust in institutions, especially among conservative or Black respondents.^30^^,^^31^ Trusted messengers, such as community and organizational leaders and trusted healthcare practitioners,^21^^,^^22^ can also tailor vaccine communications to increase transparency and address specific community concerns. For example, healthcare practitioners could provide detailed vaccine safety data to address lingering safety concerns regarding the vaccines. For groups where mistrust was more salient, strong relationships should be built between trusted leaders and their communities to address the lack of trust in institutions that develop or recommend the vaccines.^35^

Future research should investigate the varied types of lingering hesitancy, including hesitancy that arises from safety concerns versus a lack of trust in institutions. It is also worth exploring the timing of lingering hesitancy. For instance, does it exist before an individual receives a vaccine and then re-emerges before the next dose, or is it generated in response to the vaccine experience?

### Limitations

This study has several limitations. First, the authors used a cross-sectional survey to collect data during the COVID-19 pandemic, when policies and recommendations were changing rapidly. Consequently, the authors can only suggest relationships between variables and cannot investigate causation. In the future, longitudinal data would be useful for assessing cause-and-effect relationships between the constructs and the timing of lingering hesitancy. Second, the authors collaborated with citizen scientists from Chicago CBOs and a rural county health department to develop the survey and recruit a mix of participants from areas most impacted by COVID-19. However, each site used varied recruitment methods, which might have influenced the survey results.

Third, the study sample was more employed and educated than the general population in Illinois, which might have skewed perceptions of the COVID-19 vaccines. The authors used a convenience sample collected in collaboration with community partners. Therefore, these results apply to rural and urban communities in Northern Illinois. Future studies are needed to assess the generalizability of these findings. Despite these limitations, this study shows significant variations in COVID-19 vaccine attitudes and safety concerns within a vaccinated sample. Future research is needed to compare findings across other contexts and examine the extent to which these results hold. Such studies could compare similar surveys across diverse geographic locations and examine the factors associated with messages or trusted messengers that alleviate vaccinated individuals’ concerns.

## CONCLUSIONS

Most public health efforts aimed at COVID-19 vaccine outreach and messaging have focused on increasing vaccine uptake, thus targeting unvaccinated individuals.^32^^,^^33^ As the focus shifts toward annual COVID-19 vaccinations,^14^^,^^15^ it is essential to consider lingering hesitancy among those who have received previous vaccine doses.^34^ Trusted messengers are essential partners in understanding and communicating the reasons for community-level vaccine hesitancy^35^ and delivering tailored information to target communities.

## Appendix Supplementary materials

Image, application 1

## Footnotes

Supplementary material associated with this article can be found in the online version at doi:10.1016/j.focus.2025.100437.

## Footnotes

a During data collection, the research team noticed unusual “bot-enabled” survey activity. Other researchers doing COVID-19 research at the same time reported similar experiences. The authors worked with other institutions to develop a systematic process for identifying and excluding suspicious data. One critical way was by comparing start times, end times, or completion times in Qualtrics. Those with identical data were often removed.

b For some questions, participants were given the option to select “I’m not sure.” On the Likert scales, the neutral response category “Neither agree nor disagree” was assigned a score of 3, whereas the response category “I’m not sure” was assigned the item’s mean score. Replacing “I’m not sure” with the mean value allowed the authors to retain power and use a value that represents the spread of scores for the corresponding survey item.

c Participants identifying as Asian, Native American/ Indian, Alaska Native/ Hawaiian, or identifying with two or more races were excluded from the intergroup comparisons due to a small sample size.
