# Supplementary material for: Lingering Hesitancy: Persistent Uncertainty About the COVID-19 Vaccines Among Previously Vaccinated Individuals
Source: AJPM Focus. 2025 Sep 5;5(1):100437. doi: 10.1016/j.focus.2025.100437 (PMC12769783; doi:10.1016/j.focus.2025.100437)
Supplement: Supplementary file 1 [file mmc1.docx]

**Online Appendix**

**Supplemental Tables**

**Appendix Table 1**: Factor structure and item loadings.

| **(1=strongly agree, 5= strongly disagree)** | **Factor loadings** | **Cronbach’s alpha (Raw**) |
| --- | --- | --- |
| **COVID-19 Vaccine Attitudes** **(n=9 items)** |  | **0.878** |
| The COVID vaccine reduces the spread of the COVID-19 virus | 0.495 |  |
| I wear masks when they are recommended | 0.543 |  |
| Getting a COVID-19 vaccine is necessary even if a person has already had COVID-19 | 0.600 |  |
| ***I would be more likely to get a COVID-19 vaccine or booster:*** |  |  |
| If I could get the vaccine near my home or work | 0.724 |  |
| If my workplace gave employees time off to get vaccinated or made it easy to get the vaccine (e.g. vaccinations on site) | 0.748 |  |
| If it was required by my employer or places where people congregate (airport, music venue, etc.) | 0.636 |  |
| If people in my household were at high risk of getting seriously ill | 0.714 |  |
| If I already had COVID-19 | 0.572 |  |
| If a deadlier variant was common where I live | 0.703 |  |
| **Mistrust in government and institutions (n=4 items)** |  | **0.723** |
| I trust that the government is making decisions in my best interest with respect to the COVID-19 vaccine | 0.605 |  |
| I trust information and decisions about COVID-19 provided by my local health department (e.g., Chicago Department of Public Health or Stephenson County Health Department) | 0.441 |  |
| I trust the drug companies manufacturing the COVID-19 vaccines | 0.572 |  |
| Getting a COVID-19 vaccine should be mandatory for adults | 0.444 |  |
| **COVID-19 Vaccine Safety Concerns (n=5 items)**  **(*1=strongly disagree, 5= strongly agree)*** |  | **0.610** |
| The COVID-19 vaccines were developed too fast | 0.405 |  |
| There is not enough data about the safety of the COVID-19 vaccine | 0.489 |  |
| The best way to solve the COVID-19 crisis is for most people to get the disease, then we will have herd immunity in the future | 0.459 |  |
| I do not have to get vaccinated because others around me are vaccinated against COVID-19 | 0.426 |  |
| My spiritual/religious beliefs stop me from getting any of the COVID-19 vaccines | 0.479 |  |
| **Barriers toward accessing the COVID vaccines (n=10 items)**  ***(1=strongly disagree, 5= strongly agree)*** |  | **0.845** |
| ***I would be less likely to get a COVID-19 vaccine or booster:*** |  |  |
| If I had a hard time making an appointment online or over the phone | 0.722 |  |
| If I had a hard time getting an appointment that works with my schedule | 0.781 |  |
| If I had to wait in line for a long time to get the vaccine | 0.724 |  |
| If my doctor or health care provider did not specifically recommend it for me | 0.504 |  |
| If I had to show proof of health insurance to get the COVID-19 vaccine | 0.578 |  |
| If I had to show proof of citizenship | 0.558 |  |
| If I had to travel a long distance to get a vaccine or had other transportation issues | 0.696 |  |
| If people were pressuring me to vaccinated | 0.530 |  |
| If people I take care of would suffer from me getting bad side effects from a vaccine | 0.421 |  |
| If I had side effects from a previous vaccine | 0.427 |  |

**Appendix Table 2**: Factor mean scores and inter-factor correlations.

|  | **N** | **Mean (SD)** | **Range** | **1. Negative Attitudes** | **2. Barriers** | **3. Mistrust** | **4. Safety**  **Concerns** |
| --- | --- | --- | --- | --- | --- | --- | --- |
| **1. Negative Attitudes** | 523 | 2.20 (0.81) | 1-5 |  |  |  |  |
| **2. Barriers** | 539 | 3.05 (0.76) | 1-5 | -0.01 |  |  |  |
| **3. Mistrust** | 553 | 2.64 (0.86) | 1-5 | 0.61 | 0.17 |  |  |
| **4. Safety**  **Concerns** | 555 | 2.46 (0.71) | 1-5 | 0.37 | 0.22 | 0.32 |  |

*Note*: Numbers highlighted in blue represent correlation coefficients between each pair of constructs. For example, the correlation coefficient between Negative Attitudes and Mistrust was 0.61.

**Appendix Table 3**: Summary of vaccine uptake among respondents in our sample

| **Respondent description** | **n (col %)** |
| --- | --- |
| **1) Received primary series of vaccines** *(i.e 2 shots of Moderna/Pfizer or 1 shot of Johnson & Johnson)* | 537 (95.9) |
| **2) Received only 1 shot of Moderna/Pfizer** | 23 (4.1) |
| **Total** | **560 (100)** |
| **1) Respondents willing to receive a booster** | 422 (76.0) |
| **2) Not willing to receive a booster** | 81 (14.1) |
| **3) Unsure about receiving a booster** | 57 (9.8) |
| **Total** | **560 (100)** |

**Appendix Table 4**: Analysis of individual survey items across Constructs.

| **CONSTRUCT** | **SAFETY CONCERNS** | **SAFETY CONCERNS** | **NEGATIVE ATTITUDES** | **NEGATIVE ATTITUDES** | **MISTRUST** | **MISTRUST** |
| --- | --- | --- | --- | --- | --- | --- |
| **ITEM** | The COVID-19 vaccines were developed too fast. | There is not enough data about the safety of the COVID-19 vaccine. | The best way to solve the COVID-19 crisis is for most people to get the disease, then we will have herd immunity in the future | Getting a COVID-19 vaccine is necessary even if a person has already had COVID-19 [Disagree] | I trust that the government is making decisions in my best interest with respect to the COVID-19 vaccine [Disagree] | I trust the drug companies manufacturing the COVID-19 vaccines [Disagree] |
| **n (%)** | | | | | | |
| Total | **198 (36.5)***** | **155 (28.8)***** | **152 (28.6)***** | **128 (23.8)***** | **138 (25.8)***** | **123 (22.7)***** |
| **Residence** | | | | | | |
| Rural | 19 (16.4) | 26 (22.6) | 30 (25.9) | 15 (12.8) | 22 (18.6) | 18 (15.0) |
| Urban | **179 (41.9)***** | 129 (30.4) | 122 (29.3) | **113 (26.9)*** | 116 (27.8) | 105 (24.9) |
| **Political Affiliation** | | | | | | |
| Conservative | 39 (35.1) | 35 (31.8) | 30 (27.0) | **48 (43.2)***** | **35 (31.0)***** | **28 (25.2)*** |
| Liberal | 69 (33.7) | 47 (23.0) | 50 (24.3) | 40 (19.2) | 40 (19.7) | 46 (21.8) |
| Moderate | **50 (42.4)**** | 34 (29.3) | 42 (36.5)* | 24 (21.2) | 28 (23.7) | 25 (21.4) |
| Prefer not to answer | 23 (33.3) | **29 (42.0)***** | 18 (27.7) | 5 (7.5) | 20 (29.0) | 15 (21.7) |
| **Race/Ethnicity** | | | | | | |
| Black | **49 (58.3)**** | 30 (34.9) | 26 (32.1) | 18 (22.2) | 15 (19.0) | 13 (16.3) |
| White | 81 (30.9) | 70 (27.1) | 69 (26.3) | **73 (28.2)**** | 60 (22.7) | 60 (22.8) |
| Hispanic | 55 (34.6) | 44 (28.0) | 48 (31.4) | 23 (14.5) | **51 (32.9)*** | **41 (25.6)*** |

*Note*: Frequencies (n) and percentages (%) are based on those who agreed with the statement. Items with a [Disagree] represent those who disagreed with the statement. Significant differences were assessed using the chi-square test of independence. *p<.05, **p<.01, ***p<.001.

**Appendix Table 5**: Reported workplace mandates between urban and rural participants

| *Does your workplace require you to be vaccinated?* | | | |
| --- | --- | --- | --- |
|  | **Urban (n, col %)** | **Rural (n, col %)** | ***P**** |
| **Yes** | 302 (69.3) | 40 (33.3) | <.000^a^ |
| **No** | 92 (21.1) | 76 (63.3) |  |
| **I’m not sure** | 42 (9.6) | 4 (3.4) |  |
| **Total** | 436 (100) | 120 (100) |  |

^*^Significance assessed using the chi-square test of independence. ^a^*X*^2^ = 51.3, DF=1, V=0.3

**Appendix Tables 6-7**: Bootstrapping analysis showing the mediating effect of mistrust on the relationship between contextual variables and lingering hesitancy. *NB: individuals residing in urban, individuals who identified as white, individuals who identified as liberal, and those who were willing to take a booster were set as reference groups. Results in bold were statistically significant.*

**Appendix Table 6: Context🡪Mistrust🡪Negative Attitudes**

| **Variable** | **Indirect Effect** | **Direct Effect** | **Total Effect** |
| --- | --- | --- | --- |
| **Residence** | | | |
| Rural | -0.05  (-0.13, 0.03) | **-0.40**  **(-0.53, -0.27)** | **-0.45**  **(-0.60, -0.30)** |
| **Political Affiliation** | | | |
| Conservative | **0.10**  **(0.01, 0.18)** | **0.27**  **(0.13, 0.42)** | **0.37**  **(0.21, 0.54)** |
| Moderate | -0.00  (-0.09, 0.08) | -0.03  (-0.16, 0.11) | -0.03  (-0.19, 0.13) |
| Preferred not to answer | **0.10**  **(0.01, 0.20)** | -0.16  (-0.32, 0.01) | -0.05  (-0.24, 0.14) |
| **Race/Ethnicity** | | | |
| Black/African American | **-0.13**  **(-0.22,-0.05)** | **-0.22**  **(-0.38, -0.07)** | **-0.35**  **(-0.53, -0.18)** |
| Hispanic | -0.06  (-0.15, 0.03) | **-0.34**  **(-0.47, -0.21)** | **-0.40**  **(-0.55, -0.25)** |
| **Intention to Take a Booster** | | | |
| Not willing | **0.49**  **(0.37, 0.61)** | **0.59**  **(0.41, 0.76)** | **1.07**  **(0.90, 1.26)** |
| Not sure | **0.31**  **(0.20, 0.42)** | **0.20**  **(0.02, 0.38)** | **0.51**  **(0.30, 0.72)** |

**Appendix Table 7: Context🡪Mistrust🡪Safety Concerns**

| **Variable** | **Indirect Effect** | **Direct Effect** | **Total Effect** |
| --- | --- | --- | --- |
| **Residence** | | | |
| Rural | -0.02  (-0.05, 0.01) | **-0.35**  **(-0.50, -0.20)** | **-0.37**  **(-0.51, -0.22)** |
| **Political Affiliation** | | | |
| Conservative | **0.03**  **(0.00, 0.08)** | **0.18**  **(0.02, 0.34)** | **0.21**  **(0.05, 0.37)** |
| Moderate | -0.01  (-0.04, 0.03) | 0.14  (-0.01, 0.29) | 0.13  (-0.02, 0.28) |
| Preferred not to answer | 0.03  (-0.00, 0.08) | **0.20**  **(0.03, 0.38)** | **0.24**  **(0.05, 0.42)** |
| **Race/Ethnicity** | | | |
| Black/African American | **-0.05**  **(-0.09, -0.01)** | 0.15  (-0.02, 0.32) | 0.10  (-0.07, 0.27) |
| Hispanic | -0.02  (-0.05, 0.01) | -0.09  (-0.23, 0.05) | -0.11  (-0.25, 0.04) |
| **Intention to Take a Booster** | | | |
| Not willing | **0.18**  **(0.08, 0.29)** | **0.42**  **(0.23, 0.62)** | **0.60**  **(0.42, 0.78)** |
| Not sure | **0.12**  **(0.05, 0.20)** | **0.23**  **(0.03, 0.43)** | **0.35**  **(0.15, 0.55)** |

**Appendix Table 8**: Additional models showing associations between the contextual variables and lingering hesitancy.

|  | **Outcome 1** | | **Outcome 2** | |
| --- | --- | --- | --- | --- |
|  | **7^a^**  **Negative Vaccine Attitudes**  **(barriers)** | **8**  **Negative Vaccine Attitudes (mistrust & barriers)** | **9**  **Vaccine Safety Concerns (barriers)** | **10**  **Vaccine**  **Safety**  **Concerns**  **(mistrust & barriers)** |
| Intercept | **2.29*****  **(2.03, 2.55)** | **1.36*****  **(1.09, 1.62)** | **1.83*****  **(1.58, 2.07)** | **1.51*****  **(1.22, 1.80)** |
| **Residence** | | |  | |
| Rural | **-0.46*****  **(-0.61, -0.30)** | **-0.41*****  **(-0.54, -0.28)** | **-0.36*****  **(-0.50, -0.21)** | **-0.34*****  **(-0.48, -0.20)** |
| Urban | --- | --- | --- | --- |
| **Political Affiliation** | | |  | |
| Preferred not to answer | -0.01  (-0.21, 0.18) | -0.10  (-0.27, 0.06) | **0.20***  **(0.02, 0.38)** | 0.17  (-0.00, 0.35) |
| Moderate | -0.03  (-0.19, 0.13) | -0.02  (-0.15, 0.12) | 0.07  (-0.08, 0.22) | 0.08  (-0.06, 0.23) |
| Conservative | **0.38*****  **(0.21, 0.55)** | 0.29  (0.15, 0.43) | **0.20***  **(0.04, 0.35)** | **0.17***  **(0.01, 0.33)** |
| Liberal | --- | --- | --- | --- |
| **Race/Ethnicity** | | |  | |
| Black/African American | **-0.34*****  **(-0.52, -0.16)** | **-0.23****  **(-0.38, -0.08)** | 0.08  (-0.09, 0.25) | 0.12  (-0.05, 0.29) |
| Hispanic | **-0.41*****  **(-0.56, -0.26)** | **-0.34*****  **(-0.47, -0.21)** | **-0.15***  **(-0.29, 0.00)** | -0.13  (-0.27, 0.01) |
| White | --- | --- | --- | --- |
| **Intention to Take a Booster** | | |  | |
| Not sure | **0.54*****  **(0.33, 0.75)** | **0.24***  **(0.05, 0.42)** | **0.33****  **(0.13, 0.53)** | **0.23***  **(0.02, 0.43)** |
| Not willing | **1.09*****  **(0.90, 1.27)** | **0.60*****  **(0.43, 0.77)** | **0.60*****  **(0.42, 0.77)** | **0.44*****  **(0.24, 0.63)** |
| Willing | --- | --- | --- | --- |
| **Mistrust** |  | **0.45***** |  | **0.15***** |
| **Barriers** | -0.03 | **-0.09*** | **0.17***** | **0.16***** |
| R^2^ | 0.34 | 0.52 | 0.21 | 0.24 |
| *p* | <0.001 | <0.000 | <0.001 | <0.000 |
| *F-value* | 25.49 | 50.15 | 13.63 | 14.21 |

Note: *Urban*, *Liberal*, *White*, and *Willing to take a booster* were set as reference groups for comparison and thus omitted from the table. *p<.05, **p<.01, ***p<.001. **^a^** Numbers denote the model number as referenced in the narrative.

**Appendix Tables 9-10**: Bootstrapping analysis showing the mediating effect of barriers on the relationship between contextual variables and lingering hesitancy. *NB: individuals residing in urban, individuals who identified as white, individuals who identified as liberal, and those who were willing to take a booster were set as reference groups. Results in bold were statistically significant.*

Appendix Table 9: **Context🡪Barriers🡪Negative Attitudes**

| **Variable** | **Indirect Effect** | **Direct Effect** | **Total Effect** |
| --- | --- | --- | --- |
| **Residence** | | | |
| Rural | 0.00  (-0.01, 0.01) | **-0.46**  **(-0.61, -0.30)** | **-0.46**  **(-0.61, -0.30)** |
| **Political Affiliation** | | | |
| Conservative | -0.01  (-0.03, 0.01) | **0.38**  **(0.21, 0.55)** | **0.37**  **(0.21, 0.54)** |
| Moderate | -0.01  (-0.03, 0.01) | -0.03  (-0.19, 0.13) | -0.04  (-0.20, 0.12) |
| Preferred not to answer | -0.01  (-0.05, 0.02) | -0.01  (-0.21, 0.18) | -0.02  (-0.22, 0.17) |
| **Race/Ethnicity** | | | |
| Black/African American | 0.00  (-0.01, 0.02) | **-0.34**  **(-0.52, -0.16)** | **-0.34**  **(-0.52, -0.16)** |
| Hispanic | 0.01  (-0.03, 0.01) | **-0.41**  **(-0.56, -0.26)** | **-0.42**  **(-0.57, -0.27)** |
| **Intention to Take a Booster** | | | |
| Not willing | -0.00  (-0.02, 0.01) | **1.09**  **(0.90, 1.27)** | **1.08**  **(0.90, 1.27)** |
| Not sure | -0.01  (-0.04, 0.02) | **0.54**  **(0.33, 0.75)** | **0.53**  **(0.32, 0.74)** |

Appendix Table 10: **Context🡪Barriers🡪Safety Concerns**

| **Variable** | **Indirect Effect** | **Direct Effect** | **Total Effect** |
| --- | --- | --- | --- |
| **Residence** | | | |
| Rural | -0.00  (-0.04, 0.03) | **-0.36**  **(-0.50, -0.21)** | **-0.37**  **(-0.51, -0.22)** |
| **Political Affiliation** | | | |
| Conservative | 0.03  (-0.00, 0.07) | **0.20**  **(0.04, 0.35)** | **0.21**  **(0.05, 0.37)** |
| Moderate | **0.04**  **(0.01, 0.08)** | 0.07  (-0.08, 0.22) | 0.13  (-0.02, 0.28) |
| Preferred not to answer | **0.07**  **(0.01, 0.13)** | **0.20**  **(0.02, 0.38)** | **0.24**  **(0.05, 0.42)** |
| **Race/Ethnicity** | | | |
| Black/African American | -0.02  (-0.05, 0.01) | 0.08  (-0.09, 0.25) | 0.10  (-0.07, 0.27) |
| Hispanic | **0.03**  **(0.00, 0.07)** | **-0.15**  **(-0.29, 0.00)** | -0.11  (-0.25, 0.04) |
| **Intention to Take a Booster** | | | |
| Not willing | 0.02  (-0.02, 0.07) | **0.60**  **(0.42, 0.77)** | **0.60**  **(0.42, 0.78)** |
| Not sure | 0.04  (-0.00, 0.10) | **0.33**  **(0.13, 0.53)** | **0.35**  **(0.15, 0.55)** |
